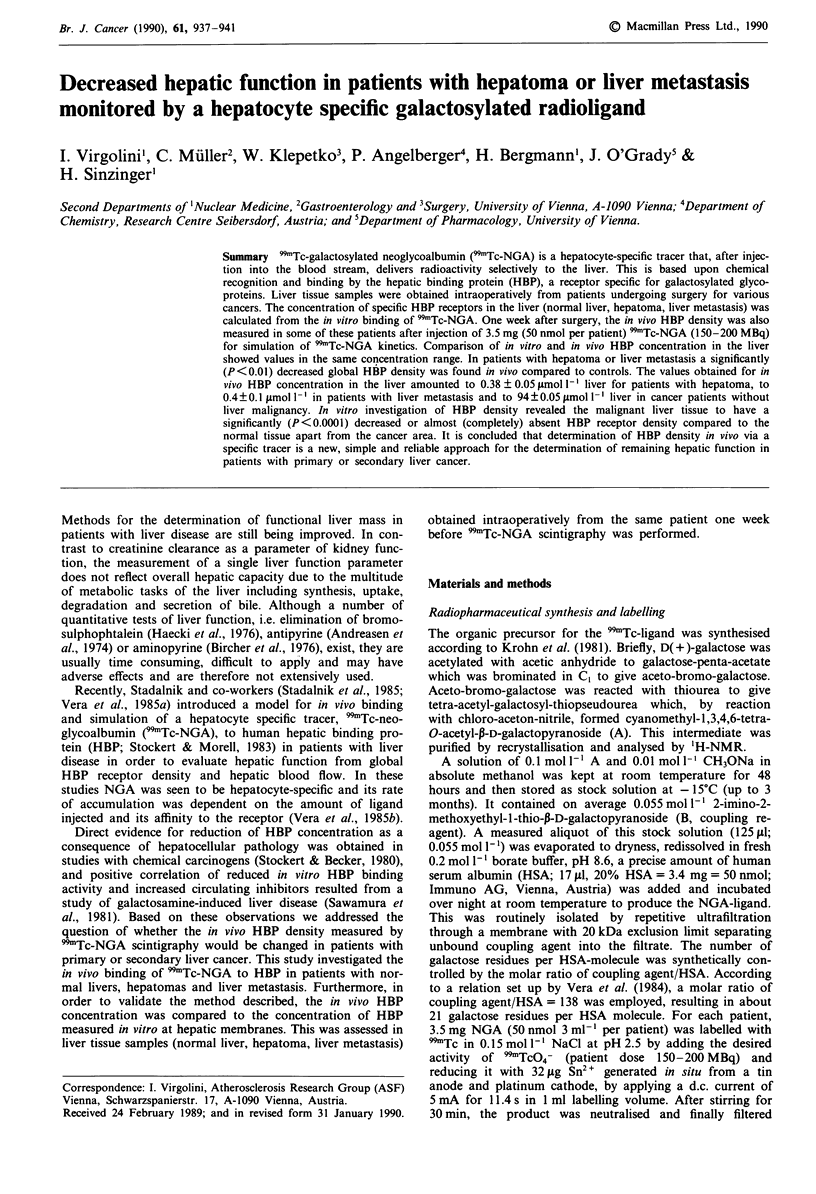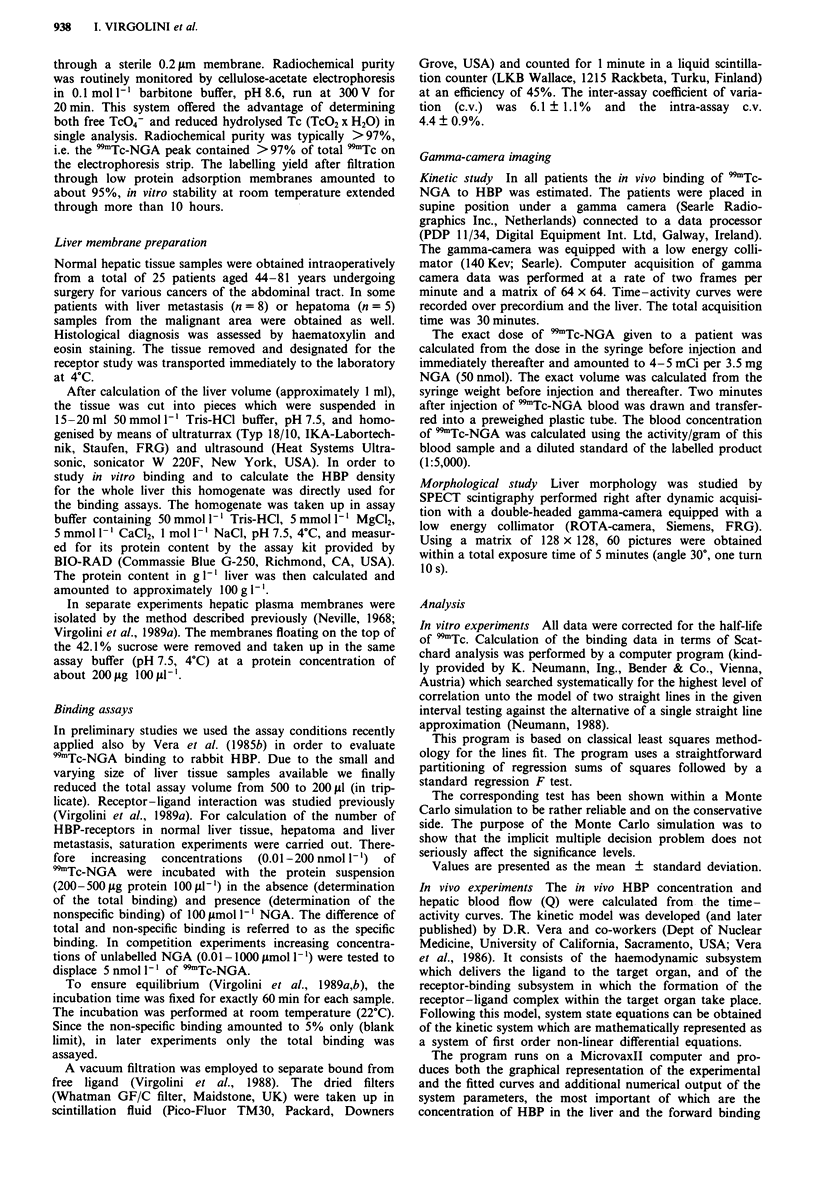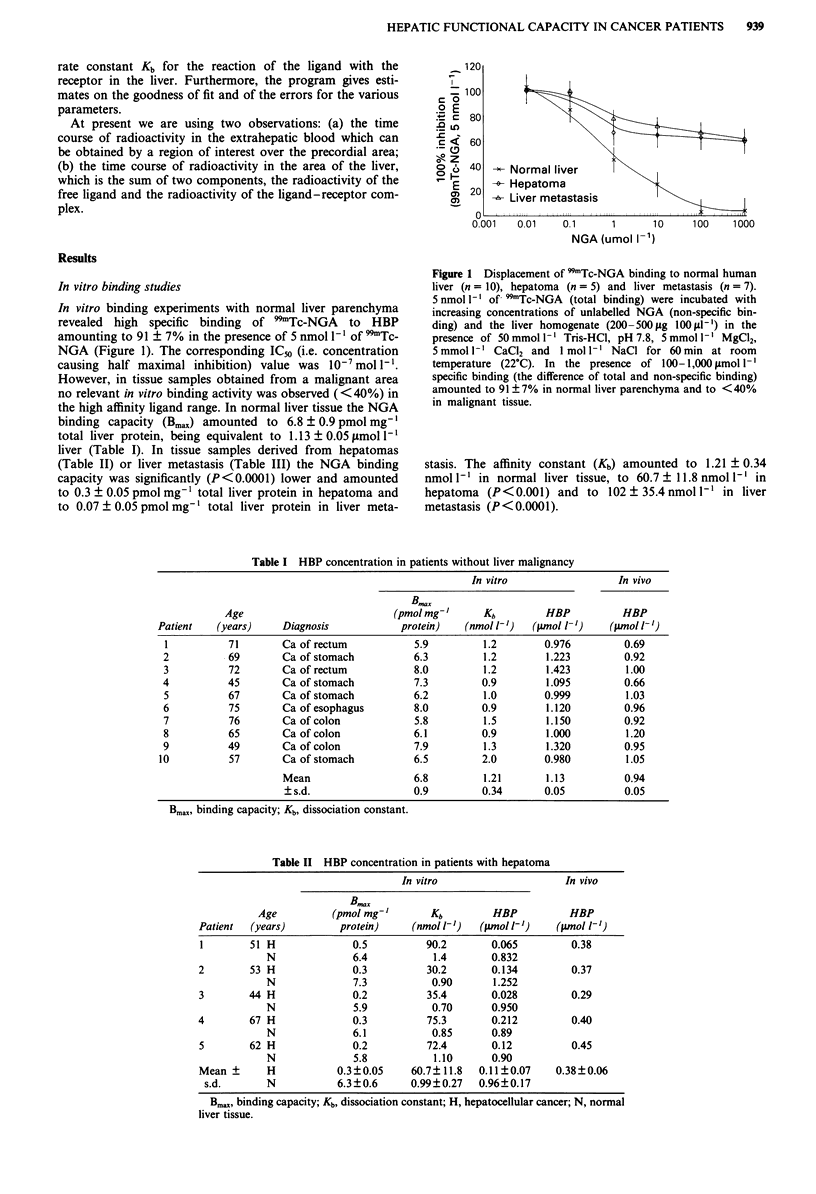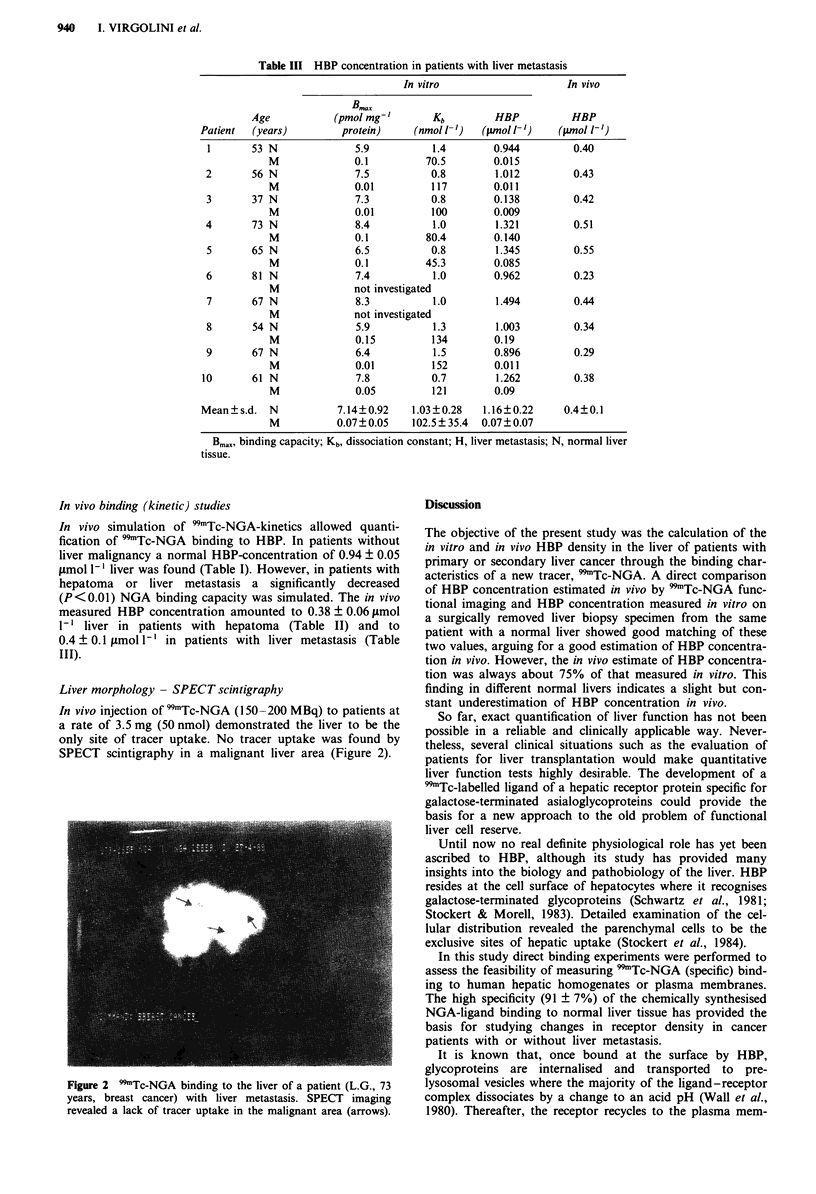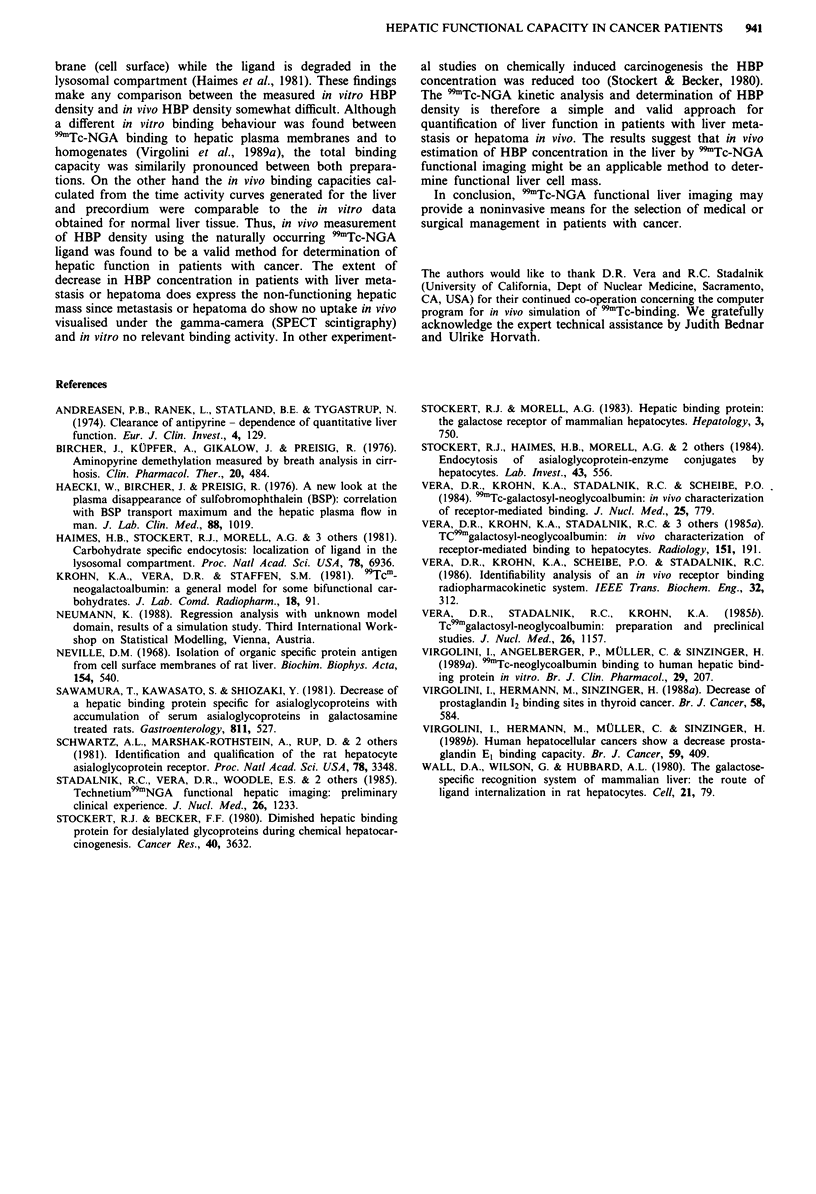# Decreased hepatic function in patients with hepatoma or liver metastasis monitored by a hepatocyte specific galactosylated radioligand.

**DOI:** 10.1038/bjc.1990.210

**Published:** 1990-06

**Authors:** I. Virgolini, C. Müller, W. Klepetko, P. Angelberger, H. Bergmann, J. O'Grady, H. Sinzinger

**Affiliations:** Second Department of Nuclear Medicine, University of Vienna, Austria.

## Abstract

**Images:**


Br. J. Cancer (1990), 61, 937-941 © Macmillan Press Ltd., 1990

### Decreased hepatic function in patients with hepatoma or liver metastasis monitored by a hepatocyte specific galactosylated radioligand

I. Virgolini[1], C. Müller[2], W. Klepetko[3], P. Angelberger[4], H. Bergmann[1], J. O'Grady[5] & H. Sinzinger[1]

*Second Departments of [1]Nuclear Medicine, [2]Gastroenterology and [3]Surgery, University of Vienna, A-1090 Vienna; [4]Department of Chemistry, Research Centre Seibersdorf, Austria; and [5]Department of Pharmacology, University of Vienna.*

**Summary** $^{99m}$Tc-galactosylated neoglycoalbumin ($^{99m}$Tc-NGA) is a hepatocyte-specific tracer that, after injection into the blood stream, delivers radioactivity selectively to the liver. This is based upon chemical recognition and binding by the hepatic binding protein (HBP), a receptor specific for galactosylated glycoproteins. Liver tissue samples were obtained intraoperatively from patients undergoing surgery for various cancers. The concentration of specific HBP receptors in the liver (normal liver, hepatoma, liver metastasis) was calculated from the *in vitro* binding of $^{99m}$Tc-NGA. One week after surgery, the *in vivo* HBP density was also measured in some of these patients after injection of 3.5 mg (50 nmol per patient) $^{99m}$Tc-NGA (150-200 MBq) for simulation of $^{99m}$Tc-NGA kinetics. Comparison of *in vitro* and *in vivo* HBP concentration in the liver showed values in the same concentration range. In patients with hepatoma or liver metastasis a significantly ($P<0.01$) decreased global HBP density was found *in vivo* compared to controls. The values obtained for *in vivo* HBP concentration in the liver amounted to $0.38 \pm 0.05\ \mu mol\ l^{-1}$ liver for patients with hepatoma, to $0.4 \pm 0.1\ \mu mol\ l^{-1}$ in patients with liver metastasis and to $94 \pm 0.05\ \mu mol\ l^{-1}$ liver in cancer patients without liver malignancy. *In vitro* investigation of HBP density revealed the malignant liver tissue to have a significantly ($P<0.0001$) decreased or almost (completely) absent HBP receptor density compared to the normal tissue apart from the cancer area. It is concluded that determination of HBP density *in vivo* via a specific tracer is a new, simple and reliable approach for the determination of remaining hepatic function in patients with primary or secondary liver cancer.

Methods for the determination of functional liver mass in patients with liver disease are still being improved. In contrast to creatinine clearance as a parameter of kidney function, the measurement of a single liver function parameter does not reflect overall hepatic capacity due to the multitude of metabolic tasks of the liver including synthesis, uptake, degradation and secretion of bile. Although a number of quantitative tests of liver function, i.e. elimination of bromosulphophtalein (Haecki *et al.*, 1976), antipyrine (Andreasen *et al.*, 1974) or aminopyrine (Bircher *et al.*, 1976), exist, they are usually time consuming, difficult to apply and may have adverse effects and are therefore not extensively used.

Recently, Stadalnik and co-workers (Stadalnik *et al.*, 1985; Vera *et al.*, 1985*a*) introduced a model for *in vivo* binding and simulation of a hepatocyte specific tracer, $^{99m}$Tc-neoglycoalbumin ($^{99m}$Tc-NGA), to human hepatic binding protein (HBP; Stockert & Morell, 1983) in patients with liver disease in order to evaluate hepatic function from global HBP receptor density and hepatic blood flow. In these studies NGA was seen to be hepatocyte-specific and its rate of accumulation was dependent on the amount of ligand injected and its affinity to the receptor (Vera *et al.*, 1985*b*).

Direct evidence for reduction of HBP concentration as a consequence of hepatocellular pathology was obtained in studies with chemical carcinogens (Stockert & Becker, 1980), and positive correlation of reduced *in vitro* HBP binding activity and increased circulating inhibitors resulted from a study of galactosamine-induced liver disease (Sawamura *et al.*, 1981). Based on these observations we addressed the question of whether the *in vivo* HBP density measured by $^{99m}$Tc-NGA scintigraphy would be changed in patients with primary or secondary liver cancer. This study investigated the *in vivo* binding of $^{99m}$Tc-NGA to HBP in patients with normal livers, hepatomas and liver metastasis. Furthermore, in order to validate the method described, the *in vivo* HBP concentration was compared to the concentration of HBP measured *in vitro* at hepatic membranes. This was assessed in liver tissue samples (normal liver, hepatoma, liver metastasis) obtained intraoperatively from the same patient one week before $^{99m}$Tc-NGA scintigraphy was performed.

Correspondence: I. Virgolini, Atherosclerosis Research Group (ASF) Vienna, Schwarzspanierstr. 17, A-1090 Vienna, Austria.
Received 24 February 1989; and in revised form 31 January 1990.

### Materials and methods

### *Radiopharmaceutical synthesis and labelling*

The organic precursor for the $^{99m}$Tc-ligand was synthesised according to Krohn *et al.* (1981). Briefly, D(+)-galactose was acetylated with acetic anhydride to galactose-penta-acetate which was brominated in $C_1$ to give aceto-bromo-galactose. Aceto-bromo-galactose was reacted with thiourea to give tetra-acetyl-galactosyl-thiopseudourea which, by reaction with chloro-aceton-nitrile, formed cyanomethyl-1,3,4,6-tetra-*O*-acetyl-β-D-galactopyranoside (A). This intermediate was purified by recrystallisation and analysed by $^1$H-NMR.

A solution of $0.1\ mol\ l^{-1}$ A and $0.01\ mol\ l^{-1}$ $CH_3ONa$ in absolute methanol was kept at room temperature for 48 hours and then stored as stock solution at $-15°C$ (up to 3 months). It contained on average $0.055\ mol\ l^{-1}$ 2-imino-2-methoxyethyl-1-thio-β-D-galactopyranoside (B, coupling reagent). A measured aliquot of this stock solution (125 µl; $0.055\ mol\ l^{-1}$) was evaporated to dryness, redissolved in fresh $0.2\ mol\ l^{-1}$ borate buffer, pH 8.6, a precise amount of human serum albumin (HSA; 17 µl, 20% HSA = 3.4 mg = 50 nmol; Immuno AG, Vienna, Austria) was added and incubated over night at room temperature to produce the NGA-ligand. This was routinely isolated by repetitive ultrafiltration through a membrane with 20 kDa exclusion limit separating unbound coupling agent into the filtrate. The number of galactose residues per HSA-molecule was synthetically controlled by the molar ratio of coupling agent/HSA. According to a relation set up by Vera *et al.* (1984), a molar ratio of coupling agent/HSA = 138 was employed, resulting in about 21 galactose residues per HSA molecule. For each patient, 3.5 mg NGA ($50\ nmol\ 3\ ml^{-1}$ per patient) was labelled with $^{99m}$Tc in $0.15\ mol\ l^{-1}$ NaCl at pH 2.5 by adding the desired activity of $^{99m}TcO_4^-$ (patient dose 150-200 MBq) and reducing it with 32 µg $Sn^{2+}$ generated *in situ* from a tin anode and platinum cathode, by applying a d.c. current of 5 mA for 11.4 s in 1 ml labelling volume. After stirring for 30 min, the product was neutralised and finally filtered through a sterile 0.2 μm membrane. Radiochemical purity was routinely monitored by cellulose-acetate electrophoresis in 0.1 mol $l^{-1}$ barbitone buffer, pH 8.6, run at 300 V for 20 min. This system offered the advantage of determining both free $TcO_4^-$ and reduced hydrolysed Tc ($TcO_2$ x $H_2O$) in single analysis. Radiochemical purity was typically >97%, i.e. the $^{99m}$Tc-NGA peak contained >97% of total $^{99m}$Tc on the electrophoresis strip. The labelling yield after filtration through low protein adsorption membranes amounted to about 95%, *in vitro* stability at room temperature extended through more than 10 hours.

*Liver membrane preparation*

Normal hepatic tissue samples were obtained intraoperatively from a total of 25 patients aged 44–81 years undergoing surgery for various cancers of the abdominal tract. In some patients with liver metastasis ($n = 8$) or hepatoma ($n = 5$) samples from the malignant area were obtained as well. Histological diagnosis was assessed by haematoxylin and eosin staining. The tissue removed and designated for the receptor study was transported immediately to the laboratory at 4°C.

After calculation of the liver volume (approximately 1 ml), the tissue was cut into pieces which were suspended in 15–20 ml 50 mmol $l^{-1}$ Tris-HCl buffer, pH 7.5, and homogenised by means of ultraturrax (Typ 18/10, IKA-Labortechnik, Staufen, FRG) and ultrasound (Heat Systems Ultrasonic, sonicator W 220F, New York, USA). In order to study *in vitro* binding and to calculate the HBP density for the whole liver this homogenate was directly used for the binding assays. The homogenate was taken up in assay buffer containing 50 mmol $l^{-1}$ Tris-HCl, 5 mmol $l^{-1}$ $MgCl_2$, 5 mmol $l^{-1}$ $CaCl_2$, 1 mol $l^{-1}$ NaCl, pH 7.5, 4°C, and measured for its protein content by the assay kit provided by BIO-RAD (Commassie Blue G-250, Richmond, CA, USA). The protein content in g $l^{-1}$ liver was then calculated and amounted to approximately 100 g $l^{-1}$.

In separate experiments hepatic plasma membranes were isolated by the method described previously (Neville, 1968; Virgolini *et al.*, 1989*a*). The membranes floating on the top of the 42.1% sucrose were removed and taken up in the same assay buffer (pH 7.5, 4°C) at a protein concentration of about 200 μg 100 $\mu l^{-1}$.

*Binding assays*

In preliminary studies we used the assay conditions recently applied also by Vera *et al.* (1985*b*) in order to evaluate $^{99m}$Tc-NGA binding to rabbit HBP. Due to the small and varying size of liver tissue samples available we finally reduced the total assay volume from 500 to 200 μl (in triplicate). Receptor–ligand interaction was studied previously (Virgolini *et al.*, 1989*a*). For calculation of the number of HBP-receptors in normal liver tissue, hepatoma and liver metastasis, saturation experiments were carried out. Therefore increasing concentrations (0.01–200 nmol $l^{-1}$) of $^{99m}$Tc-NGA were incubated with the protein suspension (200–500 μg protein 100 $\mu l^{-1}$) in the absence (determination of the total binding) and presence (determination of the nonspecific binding) of 100 μmol $l^{-1}$ NGA. The difference of total and non-specific binding is referred to as the specific binding. In competition experiments increasing concentrations of unlabelled NGA (0.01–1000 μmol $l^{-1}$) were tested to displace 5 nmol $l^{-1}$ of $^{99m}$Tc-NGA.

To ensure equilibrium (Virgolini *et al.*, 1989*a*,*b*), the incubation time was fixed for exactly 60 min for each sample. The incubation was performed at room temperature (22°C). Since the non-specific binding amounted to 5% only (blank limit), in later experiments only the total binding was assayed.

A vacuum filtration was employed to separate bound from free ligand (Virgolini *et al.*, 1988). The dried filters (Whatman GF/C filter, Maidstone, UK) were taken up in scintillation fluid (Pico-Fluor TM30, Packard, Downers Grove, USA) and counted for 1 minute in a liquid scintillation counter (LKB Wallace, 1215 Rackbeta, Turku, Finland) at an efficiency of 45%. The inter-assay coefficient of variation (c.v.) was 6.1 ± 1.1% and the intra-assay c.v. 4.4 ± 0.9%.

*Gamma-camera imaging*

*Kinetic study* In all patients the *in vivo* binding of $^{99m}$Tc-NGA to HBP was estimated. The patients were placed in supine position under a gamma camera (Searle Radiographics Inc., Netherlands) connected to a data processor (PDP 11/34, Digital Equipment Int. Ltd, Galway, Ireland). The gamma-camera was equipped with a low energy collimator (140 Kev; Searle). Computer acquisition of gamma camera data was performed at a rate of two frames per minute and a matrix of 64 × 64. Time–activity curves were recorded over precordium and the liver. The total acquisition time was 30 minutes.

The exact dose of $^{99m}$Tc-NGA given to a patient was calculated from the dose in the syringe before injection and immediately thereafter and amounted to 4–5 mCi per 3.5 mg NGA (50 nmol). The exact volume was calculated from the syringe weight before injection and thereafter. Two minutes after injection of $^{99m}$Tc-NGA blood was drawn and transferred into a preweighed plastic tube. The blood concentration of $^{99m}$Tc-NGA was calculated using the activity/gram of this blood sample and a diluted standard of the labelled product (1:5,000).

*Morphological study* Liver morphology was studied by SPECT scintigraphy performed right after dynamic acquisition with a double-headed gamma-camera equipped with a low energy collimator (ROTA-camera, Siemens, FRG). Using a matrix of 128 × 128, 60 pictures were obtained within a total exposure time of 5 minutes (angle 30°, one turn 10 s).

*Analysis*

*In vitro experiments* All data were corrected for the half-life of $^{99m}$Tc. Calculation of the binding data in terms of Scatchard analysis was performed by a computer program (kindly provided by K. Neumann, Ing., Bender & Co., Vienna, Austria) which searched systematically for the highest level of correlation unto the model of two straight lines in the given interval testing against the alternative of a single straight line approximation (Neumann, 1988).

This program is based on classical least squares methodology for the lines fit. The program uses a straightforward partitioning of regression sums of squares followed by a standard regression $F$ test.

The corresponding test has been shown within a Monte Carlo simulation to be rather reliable and on the conservative side. The purpose of the Monte Carlo simulation was to show that the implicit multiple decision problem does not seriously affect the significance levels.

Values are presented as the mean ± standard deviation.

*In vivo experiments* The *in vivo* HBP concentration and hepatic blood flow (Q) were calculated from the time–activity curves. The kinetic model was developed (and later published) by D.R. Vera and co-workers (Dept of Nuclear Medicine, University of California, Sacramento, USA; Vera *et al.*, 1986). It consists of the haemodynamic subsystem which delivers the ligand to the target organ, and of the receptor-binding subsystem in which the formation of the receptor–ligand complex within the target organ take place. Following this model, system state equations can be obtained of the kinetic system which are mathematically represented as a system of first order non-linear differential equations.

The program runs on a MicrovaxII computer and produces both the graphical representation of the experimental and the fitted curves and additional numerical output of the system parameters, the most important of which are the concentration of HBP in the liver and the forward binding rate constant $K_b$ for the reaction of the ligand with the receptor in the liver. Furthermore, the program gives estimates on the goodness of fit and of the errors for the various parameters.

At present we are using two observations: (a) the time course of radioactivity in the extrahepatic blood which can be obtained by a region of interest over the precordial area; (b) the time course of radioactivity in the area of the liver, which is the sum of two components, the radioactivity of the free ligand and the radioactivity of the ligand–receptor complex.

### Results

### *In vitro binding studies*

*In vitro* binding experiments with normal liver parenchyma revealed high specific binding of $^{99m}$Tc-NGA to HBP amounting to 91 ± 7% in the presence of 5 nmol $l^{-1}$ of $^{99m}$Tc-NGA (Figure 1). The corresponding $IC_{50}$ (i.e. concentration causing half maximal inhibition) value was $10^{-7}$ mol $l^{-1}$. However, in tissue samples obtained from a malignant area no relevant *in vitro* binding activity was observed (<40%) in the high affinity ligand range. In normal liver tissue the NGA binding capacity ($B_{max}$) amounted to 6.8 ± 0.9 pmol $mg^{-1}$ total liver protein, being equivalent to 1.13 ± 0.05 μmol $l^{-1}$ liver (Table I). In tissue samples derived from hepatomas (Table II) or liver metastasis (Table III) the NGA binding capacity was significantly ($P<0.0001$) lower and amounted to 0.3 ± 0.05 pmol $mg^{-1}$ total liver protein in hepatoma and to 0.07 ± 0.05 pmol $mg^{-1}$ total liver protein in liver metastasis. The affinity constant ($K_b$) amounted to 1.21 ± 0.34 nmol $l^{-1}$ in normal liver tissue, to 60.7 ± 11.8 nmol $l^{-1}$ in hepatoma ($P<0.001$) and to 102 ± 35.4 nmol $l^{-1}$ in liver metastasis ($P<0.0001$).

**Figure 1** Displacement of $^{99m}$Tc-NGA binding to normal human liver ($n = 10$), hepatoma ($n = 5$) and liver metastasis ($n = 7$). 5 nmol $l^{-1}$ of $^{99m}$Tc-NGA (total binding) were incubated with increasing concentrations of unlabelled NGA (non-specific binding) and the liver homogenate (200–500 μg 100 μ$l^{-1}$) in the presence of 50 mmol $l^{-1}$ Tris-HCl, pH 7.8, 5 mmol $l^{-1}$ $MgCl_2$, 5 mmol $l^{-1}$ $CaCl_2$ and 1 mol $l^{-1}$ NaCl for 60 min at room temperature (22°C). In the presence of 100–1,000 μmol $l^{-1}$ specific binding (the difference of total and non-specific binding) amounted to 91 ± 7% in normal liver parenchyma and to <40% in malignant tissue.

**Table I** HBP concentration in patients without liver malignancy

| | | | *In vitro* | | | *In vivo* |
|---|---|---|---|---|---|---|
| *Patient* | *Age (years)* | *Diagnosis* | *$B_{max}$ (pmol $mg^{-1}$ protein)* | *$K_b$ (nmol $l^{-1}$)* | *HBP (μmol $l^{-1}$)* | *HBP (μmol $l^{-1}$)* |
| 1 | 71 | Ca of rectum | 5.9 | 1.2 | 0.976 | 0.69 |
| 2 | 69 | Ca of stomach | 6.3 | 1.2 | 1.223 | 0.92 |
| 3 | 72 | Ca of rectum | 8.0 | 1.2 | 1.423 | 1.00 |
| 4 | 45 | Ca of stomach | 7.3 | 0.9 | 1.095 | 0.66 |
| 5 | 67 | Ca of stomach | 6.2 | 1.0 | 0.999 | 1.03 |
| 6 | 75 | Ca of esophagus | 8.0 | 0.9 | 1.120 | 0.96 |
| 7 | 76 | Ca of colon | 5.8 | 1.5 | 1.150 | 0.92 |
| 8 | 65 | Ca of colon | 6.1 | 0.9 | 1.000 | 1.20 |
| 9 | 49 | Ca of colon | 7.9 | 1.3 | 1.320 | 0.95 |
| 10 | 57 | Ca of stomach | 6.5 | 2.0 | 0.980 | 1.05 |
| | | Mean | 6.8 | 1.21 | 1.13 | 0.94 |
| | | ±s.d. | 0.9 | 0.34 | 0.05 | 0.05 |

$B_{max}$, binding capacity; $K_b$, dissociation constant.

**Table II** HBP concentration in patients with hepatoma

| | | | | *In vitro* | | *In vivo* |
|---|---|---|---|---|---|---|
| *Patient* | *Age (years)* | | *$B_{max}$ (pmol $mg^{-1}$ protein)* | *$K_b$ (nmol $l^{-1}$)* | *HBP (μmol $l^{-1}$)* | *HBP (μmol $l^{-1}$)* |
| 1 | 51 | H | 0.5 | 90.2 | 0.065 | 0.38 |
| | | N | 6.4 | 1.4 | 0.832 | |
| 2 | 53 | H | 0.3 | 30.2 | 0.134 | 0.37 |
| | | N | 7.3 | 0.90 | 1.252 | |
| 3 | 44 | H | 0.2 | 35.4 | 0.028 | 0.29 |
| | | N | 5.9 | 0.70 | 0.950 | |
| 4 | 67 | H | 0.3 | 75.3 | 0.212 | 0.40 |
| | | N | 6.1 | 0.85 | 0.89 | |
| 5 | 62 | H | 0.2 | 72.4 | 0.12 | 0.45 |
| | | N | 5.8 | 1.10 | 0.90 | |
| Mean ± | | H | 0.3±0.05 | 60.7±11.8 | 0.11±0.07 | 0.38±0.06 |
| s.d. | | N | 6.3±0.6 | 0.99±0.27 | 0.96±0.17 | |

$B_{max}$, binding capacity; $K_b$, dissociation constant; H, hepatocellular cancer; N, normal liver tissue.

**Table III** HBP concentration in patients with liver metastasis

| | | *In vitro* | | | *In vivo* |
|---|---|---|---|---|---|
| *Patient* | *Age (years)* | $B_{max}$ *(pmol mg$^{-1}$ protein)* | $K_b$ *(nmol l$^{-1}$)* | *HBP (μmol l$^{-1}$)* | *HBP (μmol l$^{-1}$)* |
| 1 | 53 N | 5.9 | 1.4 | 0.944 | 0.40 |
| | M | 0.1 | 70.5 | 0.015 | |
| 2 | 56 N | 7.5 | 0.8 | 1.012 | 0.43 |
| | M | 0.01 | 117 | 0.011 | |
| 3 | 37 N | 7.3 | 0.8 | 0.138 | 0.42 |
| | M | 0.01 | 100 | 0.009 | |
| 4 | 73 N | 8.4 | 1.0 | 1.321 | 0.51 |
| | M | 0.1 | 80.4 | 0.140 | |
| 5 | 65 N | 6.5 | 0.8 | 1.345 | 0.55 |
| | M | 0.1 | 45.3 | 0.085 | |
| 6 | 81 N | 7.4 | 1.0 | 0.962 | 0.23 |
| | M | not investigated | | | |
| 7 | 67 N | 8.3 | 1.0 | 1.494 | 0.44 |
| | M | not investigated | | | |
| 8 | 54 N | 5.9 | 1.3 | 1.003 | 0.34 |
| | M | 0.15 | 134 | 0.19 | |
| 9 | 67 N | 6.4 | 1.5 | 0.896 | 0.29 |
| | M | 0.01 | 152 | 0.011 | |
| 10 | 61 N | 7.8 | 0.7 | 1.262 | 0.38 |
| | M | 0.05 | 121 | 0.09 | |
| Mean ± s.d. | N | 7.14 ± 0.92 | 1.03 ± 0.28 | 1.16 ± 0.22 | 0.4 ± 0.1 |
| | M | 0.07 ± 0.05 | 102.5 ± 35.4 | 0.07 ± 0.07 | |

$B_{max}$, binding capacity; $K_b$, dissociation constant; H, liver metastasis; N, normal liver tissue.

### *In vivo binding (kinetic) studies*

*In vivo* simulation of $^{99m}$Tc-NGA-kinetics allowed quantification of $^{99m}$Tc-NGA binding to HBP. In patients without liver malignancy a normal HBP-concentration of 0.94 ± 0.05 μmol l$^{-1}$ liver was found (Table I). However, in patients with hepatoma or liver metastasis a significantly decreased ($P < 0.01$) NGA binding capacity was simulated. The *in vivo* measured HBP concentration amounted to 0.38 ± 0.06 μmol l$^{-1}$ liver in patients with hepatoma (Table II) and to 0.4 ± 0.1 μmol l$^{-1}$ in patients with liver metastasis (Table III).

### *Liver morphology – SPECT scintigraphy*

*In vivo* injection of $^{99m}$Tc-NGA (150–200 MBq) to patients at a rate of 3.5 mg (50 nmol) demonstrated the liver to be the only site of tracer uptake. No tracer uptake was found by SPECT scintigraphy in a malignant liver area (Figure 2).

**Figure 2** $^{99m}$Tc-NGA binding to the liver of a patient (L.G., 73 years, breast cancer) with liver metastasis. SPECT imaging revealed a lack of tracer uptake in the malignant area (arrows).

### Discussion

The objective of the present study was the calculation of the *in vitro* and *in vivo* HBP density in the liver of patients with primary or secondary liver cancer through the binding characteristics of a new tracer, $^{99m}$Tc-NGA. A direct comparison of HBP concentration estimated *in vivo* by $^{99m}$Tc-NGA functional imaging and HBP concentration measured *in vitro* on a surgically removed liver biopsy specimen from the same patient with a normal liver showed good matching of these two values, arguing for a good estimation of HBP concentration *in vivo*. However, the *in vivo* estimate of HBP concentration was always about 75% of that measured *in vitro*. This finding in different normal livers indicates a slight but constant underestimation of HBP concentration *in vivo*.

So far, exact quantification of liver function has not been possible in a reliable and clinically applicable way. Nevertheless, several clinical situations such as the evaluation of patients for liver transplantation would make quantitative liver function tests highly desirable. The development of a $^{99m}$Tc-labelled ligand of a hepatic receptor protein specific for galactose-terminated asialoglycoproteins could provide the basis for a new approach to the old problem of functional liver cell reserve.

Until now no real definite physiological role has yet been ascribed to HBP, although its study has provided many insights into the biology and pathobiology of the liver. HBP resides at the cell surface of hepatocytes where it recognises galactose-terminated glycoproteins (Schwartz *et al.*, 1981; Stockert & Morell, 1983). Detailed examination of the cellular distribution revealed the parenchymal cells to be the exclusive sites of hepatic uptake (Stockert *et al.*, 1984).

In this study direct binding experiments were performed to assess the feasibility of measuring $^{99m}$Tc-NGA (specific) binding to human hepatic homogenates or plasma membranes. The high specificity (91 ± 7%) of the chemically synthesised NGA-ligand binding to normal liver tissue has provided the basis for studying changes in receptor density in cancer patients with or without liver metastasis.

It is known that, once bound at the surface by HBP, glycoproteins are internalised and transported to prelysosomal vesicles where the majority of the ligand–receptor complex dissociates by a change to an acid pH (Wall *et al.*, 1980). Thereafter, the receptor recycles to the plasma membrane (cell surface) while the ligand is degraded in the lysosomal compartment (Haimes *et al.*, 1981). These findings make any comparison between the measured *in vitro* HBP density and *in vivo* HBP density somewhat difficult. Although a different *in vitro* binding behaviour was found between $^{99m}$Tc-NGA binding to hepatic plasma membranes and to homogenates (Virgolini *et al.*, 1989*a*), the total binding capacity was similarily pronounced between both preparations. On the other hand the *in vivo* binding capacities calculated from the time activity curves generated for the liver and precordium were comparable to the *in vitro* data obtained for normal liver tissue. Thus, *in vivo* measurement of HBP density using the naturally occurring $^{99m}$Tc-NGA ligand was found to be a valid method for determination of hepatic function in patients with cancer. The extent of decrease in HBP concentration in patients with liver metastasis or hepatoma does express the non-functioning hepatic mass since metastasis or hepatoma do show no uptake *in vivo* visualised under the gamma-camera (SPECT scintigraphy) and *in vitro* no relevant binding activity. In other experimental studies on chemically induced carcinogenesis the HBP concentration was reduced too (Stockert & Becker, 1980). The $^{99m}$Tc-NGA kinetic analysis and determination of HBP density is therefore a simple and valid approach for quantification of liver function in patients with liver metastasis or hepatoma *in vivo*. The results suggest that *in vivo* estimation of HBP concentration in the liver by $^{99m}$Tc-NGA functional imaging might be an applicable method to determine functional liver cell mass.

In conclusion, $^{99m}$Tc-NGA functional liver imaging may provide a noninvasive means for the selection of medical or surgical management in patients with cancer.

The authors would like to thank D.R. Vera and R.C. Stadalnik (University of California, Dept of Nuclear Medicine, Sacramento, CA, USA) for their continued co-operation concerning the computer program for *in vivo* simulation of $^{99m}$Tc-binding. We gratefully acknowledge the expert technical assistance by Judith Bednar and Ulrike Horvath.

### References

ANDREASEN, P.B., RANEK, L., STATLAND, B.E. & TYGASTRUP, N. (1974). Clearance of antipyrine – dependence of quantitative liver function. *Eur. J. Clin. Invest.*, **4,** 129.

BIRCHER, J., KÜPFER, A., GIKALOW, J. & PREISIG, R. (1976). Aminopyrine demethylation measured by breath analysis in cirrhosis. *Clin. Pharmacol. Ther.*, **20,** 484.

HAECKI, W., BIRCHER, J. & PREISIG, R. (1976). A new look at the plasma disappearance of sulfobromophthalein (BSP): correlation with BSP transport maximum and the hepatic plasma flow in man. *J. Lab. Clin. Med.*, **88,** 1019.

HAIMES, H.B., STOCKERT, R.J., MORELL, A.G. & 3 others (1981). Carbohydrate specific endocytosis: localization of ligand in the lysosomal compartment. *Proc. Natl Acad. Sci. USA*, **78,** 6936.

KROHN, K.A., VERA, D.R. & STAFFEN, S.M. (1981). $^{99}$Tc$^{m}$-neogalactoalbumin: a general model for some bifunctional carbohydrates. *J. Lab. Comd. Radiopharm.*, **18,** 91.

NEUMANN, K. (1988). Regression analysis with unknown model domain, results of a simulation study. Third International Workshop on Statistical Modelling, Vienna, Austria.

NEVILLE, D.M. (1968). Isolation of organic specific protein antigen from cell surface membranes of rat liver. *Biochim. Biophys. Acta*, **154,** 540.

SAWAMURA, T., KAWASATO, S. & SHIOZAKI, Y. (1981). Decrease of a hepatic binding protein specific for asialoglycoproteins with accumulation of serum asialoglycoproteins in galactosamine treated rats. *Gastroenterology*, **811,** 527.

SCHWARTZ, A.L., MARSHAK-ROTHSTEIN, A., RUP, D. & 2 others (1981). Identification and qualification of the rat hepatocyte asialoglycoprotein receptor. *Proc. Natl Acad. Sci. USA*, **78,** 3348.

STADALNIK, R.C., VERA, D.R., WOODLE, E.S. & 2 others (1985). Technetium$^{99m}$NGA functional hepatic imaging: preliminary clinical experience. *J. Nucl. Med.*, **26,** 1233.

STOCKERT, R.J. & BECKER, F.F. (1980). Dimished hepatic binding protein for desialylated glycoproteins during chemical hepatocarcinogenesis. *Cancer Res.*, **40,** 3632.

STOCKERT, R.J. & MORELL, A.G. (1983). Hepatic binding protein: the galactose receptor of mammalian hepatocytes. *Hepatology*, **3,** 750.

STOCKERT, R.J., HAIMES, H.B., MORELL, A.G. & 2 others (1984). Endocytosis of asialoglycoprotein-enzyme conjugates by hepatocytes. *Lab. Invest.*, **43,** 556.

VERA, D.R., KROHN, K.A., STADALNIK, R.C. & SCHEIBE, P.O. (1984). $^{99m}$Tc-galactosyl-neoglycoalbumin: *in vivo* characterization of receptor-mediated binding. *J. Nucl. Med.*, **25,** 779.

VERA, D.R., KROHN, K.A., STADALNIK, R.C. & 3 others (1985*a*). TC$^{99m}$galactosyl-neoglycoalbumin: *in vivo* characterization of receptor-mediated binding to hepatocytes. *Radiology*, **151,** 191.

VERA, D.R., KROHN, K.A., SCHEIBE, P.O. & STADALNIK, R.C. (1986). Identifiability analysis of an *in vivo* receptor binding radiopharmacokinetic system. *IEEE Trans. Biochem. Eng.*, **32,** 312.

VERA, D.R., STADALNIK, R.C., KROHN, K.A. (1985*b*). Tc$^{99m}$galactosyl-neoglycoalbumin: preparation and preclinical studies. *J. Nucl. Med.*, **26,** 1157.

VIRGOLINI, I., ANGELBERGER, P., MÜLLER, C. & SINZINGER, H. (1989*a*). $^{99m}$Tc-neoglycoalbumin binding to human hepatic binding protein *in vitro*. *Br. J. Clin. Pharmacol.*, **29,** 207.

VIRGOLINI, I., HERMANN, M., SINZINGER, H. (1988*a*). Decrease of prostaglandin $I_2$ binding sites in thyroid cancer. *Br. J. Cancer*, **58,** 584.

VIRGOLINI, I., HERMANN, M., MÜLLER, C. & SINZINGER, H. (1989*b*). Human hepatocellular cancers show a decrease prostaglandin $E_1$ binding capacity. *Br. J. Cancer*, **59,** 409.

WALL, D.A., WILSON, G. & HUBBARD, A.L. (1980). The galactose-specific recognition system of mammalian liver: the route of ligand internalization in rat hepatocytes. *Cell*, **21,** 79.